# Periodontal Regeneration Using Platelet-Derived Growth Factor in Infrabony Defects: A Series of Three Cases

**DOI:** 10.1155/2013/849823

**Published:** 2013-03-07

**Authors:** Kaustubh Thakare, M. L. Bhongade, Preeti Charde, Priyanka Jaiswal, Nachiket Shah, Aniruddha Deshpande

**Affiliations:** ^1^Department of Periodontology and Implantology, Sharad Pawar Dental College and Hospital, Maharashtra, Wardha 442001, India; ^2^Department of Periodontology and Implantology, CSMSS Dental College and Hospital, Maharashtra, Aurangabad 431001, India

## Abstract

The periodontal researchers and clinicians, in an effort to develop effective regenerative therapies, have sought to understand key events involved in the regeneration of periodontium. Polypeptide growth factors are a class of natural biological mediators which regulate key cellular events in tissue repair. Platelet-derived growth factor (PDGF) is the most thoroughly studied growth factor in periodontal regeneration. The present case series evaluate the effectiveness of platelet-derived growth factor (rh-PDGF-BB) in combination with beta-tricalcium phosphate (**β**-TCP) to achieve periodontal regeneration in 3 infrabony defects.

## 1. Introduction

Periodontal treatment modalities now strive to provide one or more of the following to enhance periodontal regeneration: appropriate matrices, biologic mediators, and/or precursor cells. Polypeptide growth factors are a class of natural biological mediators which regulate key cellular events in tissue repair. Platelet-derived growth factor (PDGF) is the most thoroughly studied growth factor in periodontics. Since the late 1980s, when it was first discovered that PDGF promoted regeneration of bone, cementum, and periodontal ligament (PDL) by Lynch et al. (1989) [1], nearly 100 studies have been published on its effect on PDL and alveolar bone cells and regeneration of periodontium in both animals and humans.

These studies have clearly demonstrated the mechanism of action of PDGF, showing the presence of cell surface receptors for PDGF on PDL and alveolar bone cells and elucidating PDGF's stimulatory effect on the proliferation and chemotaxis of osteoblast, PDL fibroblasts, and cementoblasts [2–4].

The presence of ideal scaffold plays a basic role in periodontal regeneration [5]. Filling the regenerative spaces with a biocompatible material may facilitate or accelerate the periodontal regeneration process by adding more solid surfaces on which the cells can initiate regeneration. Beta-tricalcium phosphate (*β*-TCP) is a purified, multicrystalline, and porous form of calcium phosphate with a Ca : Po_4_ ratio similar to that of natural bone material. It provides matrix or scaffolding for periodontal regeneration and also facilitates the stabilization of the blood clot [6].

Therefore, the present case series was carried out to evaluate the effectiveness of platelet-derived growth factor (rh-PDGF-BB) in combination with beta-tricalcium phosphate (*β*-TCP) to achieve periodontal regeneration in 3 infrabony defects.

## 2. Case Presentations

After proper examination and diagnosis, 3 patients with infrabony defect were selected from the Department of Periodontology and Implantology. Initial therapy consisting of oral hygiene instructions, supra and subgingival scaling, and root planing under local anesthesia was performed. 6 weeks after the initial therapy, surgical therapy was initiated. Prior to surgery at baseline, clinical measurements including probing pocket depth (PPD), clinical attachment loss (CAL), and gingival recession (GR) were recorded with Florida Probe (Florida Probe Corporation, Gainesville, FL, USA) with a constant probing force of 15 gm (pressure −154 N/cm^2^), tip diameter of 0.45 mm, precision of 0.2 mm and a probe length of 11 mm and William's graduated probe. At Baseline an intraoral periapical radiograph was taken of selected site with long cone (XCP Rinn, Dentsply) paralleling technique. Radiographic measurements were obtained by utilizing a film mount with millimeter grid scale (Nix Company Ltd., Tokyo, Japan). These are pocket-mount-type grids for intraoral film. Grid scale lines are printed at 1 mm intervals with bold lines at 5 mm intervals. The developed IOPA films were inserted in the mount to evaluate radiographic defect depth (DD), which is measured from the crest of bone to the base of bony defect. All the clinical and radiographic measurements were again recorded at 6 months post-operatively.

*Case  1*. A 35-year-old female patient presented with clinical and radiographic measurements on mesial surface of 46 (see Table 1).

*Case  2*. A 40-year-old male patient presented with clinical and radiographic measurements on mesial surface of 36 (see Table 2).

*Case  3*. A 38-year-old male patient presented with clinical and radiographic measurements on mesial surface of 36 (see Table 3).

### 2.1. Surgical Procedure

Immediately before the surgical treatment, the patients were made to rinse the mouth with 0.2% chlorhexidine gluconate solution for 1 minute. The Patients were draped to expose only the oral cavity. Asepsis was maintained throughout the entire procedure. The area subjected to surgery was anaesthetized by nerve block or infiltration anaesthesia, depending upon the surgical site, using local anaesthetic solution 2% solution xylocaine containing 1 : 100,000 epinephrine.

Conventional approach consisting in a periodontal access flap was initiated by intracrevicular (sulcular) incisions using Bard-Parker number 12 and 15 surgical blades on the buccal and lingual aspects. A tooth each mesial and distal to the defect associated tooth were included in the flap. 

Full-thickness (mucoperiosteal) flap was reflected using a periosteal elevator (24 G Hu-Friedy, USA) to expose alveolar bone in area of osseous defect. The osseous defect was debrided off granulation tissue using hand instruments (Gracey Curettes, Hu-Friedy, USA) and ultrasonic instruments (EMS, Minipiezon), thus exposing the root surface, alveolar bone, and periodontal ligament. Intramarrow penetration was performed with a half-round bur, in case of insufficient bleedings from the walls of the lesion. 

At this stage, the defects were treated with insertion of rh-PDGF-BB plus beta-tricalcium phosphate (GEM 21S, Growth-Factor-Enhanced Matrix, BioMimetic Therapeutics, Inc., Franklin, TN, USA). According to the manufacturer's directions, the beta-tricalcium phosphate particles were hydrated with 0.3 mg/mL rh-PDGF-BB and then gently packed into the osseous defects to the level of most coronal osseous wall. The mucoperiosteal flaps were positioned coronally to achieve primary coverage over the graft and sutured using interdental interrupted sutures (4–0, nonresorbable surgical sutures, braided black silk, Mersilk, Ethicon, Johnson Ltd.). The surgical site was dressed with periodontal surgical dressing (Coe-Pak, GC, Inc, ALSIP, IL, USA), on the buccal and lingual aspects.

Antibiotic coverage consisting in Amoxicillin 500 mg three times a day and analgesics consisting in combination of Ibuprofen 325 mg and Paracetamol 400 mg three times a day were prescribed for 5 days after surgical period. Patients were instructed to rinse twice daily with 0.2% chlorhexidine gluconate for 6 weeks. Periodontal dressing and sutures were removed 8–10 days after surgery. After this period, patients were reinstructed to resume mechanical oral hygiene measures, including careful brushing with soft toothbrush and interdental cleaning with an interdental brush) and to discontinue chlorhexidine.

In the present case series, at 6 months of followup after surgery, significant improvement was found in clinical parameters. Patients 1 and 2 showed 100% radiographic defect fill while as patient 3 showed 80% radiographic defect depth fill. *Clinical and radiographic measurements at 6 months of followup:*

*Case  1:* see Table 4.

*Case  2:* see Table 5.

*Case  3:* see Table 6.

## 3. Discussion

Changes in the clinical attachment levels (CALs) following regenerative therapy are the most commonly used clinical outcome variable in regenerative studies. In the present case series, a significantly greater amount of mean CAL gain was observed at 6 months of followup. The results obtained in the present case series are comparable with other reported studies. Camelo et al. [7] studied the effectiveness of purified recombinant human platelet-derived growth factor (rh-PDGF-BB) mixed with a synthetic *β*-TCP matrix for the treatment of osseous defects and observed significant mean CAL gain of 3.8 mm for rh-PDGF-BB combined with *β*-TCP group compared to *β*-TCP alone group (3.2 mm). Camelo et al. [8] also reported a significant mean CAL gain at sites treated with rh-PDGF-BB in combination with *β*-TCP. In the present case series, a significantly greater amount of mean CAL gain could be related to the molecular characteristic of rh-PDGF-BB that enhances cell attachment and proliferation.

**Figure 1:**
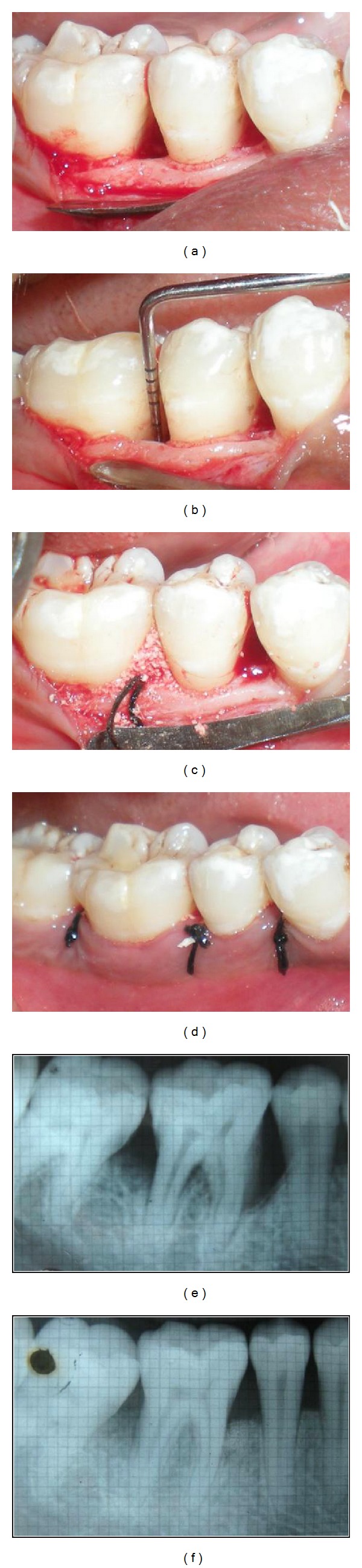
(a) Preoperative view. (b) Configuration and depth of defect. (c) Placement of rh-PDGF-BB in combination with *β*-TCP. (d) Suturing. (e) Preoperative radiograph. (f) Postoperative radiograph.

Reduction of pocket depth in order to limit the risk of local reinfection is a primary goal of periodontal therapy. Shallow pockets have a strong, negative predictive value for future disease progression, while deep pockets in treated areas are risk indicators for periodontal disease progression. In the present study, pocket depth reduction was significant in all three patients. These findings are in accordance with those of a study by Nevins et al. [9], where mean PPD reduction of 5.93 mm in sites treated with combination of rh-PDGF-BB with *β*-TCP (Figure 1).

During 6 months, the infrabony lesions in this study, responded well to rh-PDGF-BB combined with *β*-TCP treatment with regard to the reduction in radiographic defect depth. Radiographic bone measurement is a noninvasive, painless alternative to direct bone measurement. Therefore, in the present case series, radiographic monitoring of alveolar bone changes was carried out. These findings are in accordance with those of previous reported studies. Nevins et al. [10] reported a gain in defect fill of 2.9 to 3.0 mm (57%) in sites treated with rh-PDGF-BB + *β*-TCP.

## 4. Conclusion

On the basis of the results of the present case series study and within the limitations of the study, it can be concluded that treatment with rh-PDGF-BB in combination with *β*-TCP resulted in a significantly higher CAL gain and PPD reduction and radiographic defect fill.

## Figures and Tables

**Table 1:** 

PPD	CAL	GR	DD
9.8 mm	12 mm	2.2 mm	4 mm

**Table 2:** 

PPD	CAL	GR	DD
6.2 mm	6.2 mm	0 mm	3 mm

**Table 3:** 

PPD	CAL	GR	DD
5.8 mm	5.8 mm	0 mm	5 mm

**Table 4:** 

PPD	CAL	GR	DD
3.2 mm	5.4 mm	2.2 mm	0 mm

**Table 5:** 

PPD	CAL	GR	DD
3 mm	3 mm	0 mm	0 mm

**Table 6:** 

PPD	CAL	GR	DD
2.8 mm	3.6 mm	0.8 mm	1 mm
